# BSREM for Brain Metastasis Detection with 18F-FDG-PET/CT in Lung Cancer Patients

**DOI:** 10.1007/s10278-021-00570-y

**Published:** 2022-02-25

**Authors:** Virginia Liberini, Daniele A. Pizzuto, Michael Messerli, Erika Orita, Hannes Grünig, Alexander Maurer, Cäcilia Mader, Lars Husmann, Désirée Deandreis, Fotis Kotasidis, Josey Trinckauf, Alessandra Curioni, Isabelle Opitz, Sebastian Winklhofer, Martin W. Huellner

**Affiliations:** 1 Department of Nuclear Medicine, University Hospital Zürich, University of Zürich, Zürich, Switzerland; 2 Department of Medical Science, Unit of Nuclear Medicine, University of Turin, Turin, Italy; 3 Nuclear Medicine Department, S. Croce E Carle Hospital, Cuneo, Italy; 4 Nuclear Medicine Unit, Fondazione Policlinico Universitario A. Gemelli IRCCS, 00168 Rome, Italy; 5 Department of Radiology, Nippon Medical School, 1-1-5 Sendagi, Bunkyo-ku, Tokyo, 113-8603 Japan; 6 GE Healthcare, Waukesha, WI USA; 7 Department of Medical Oncology and Hematology, University Hospital Zurich, University of Zurich, Zurich, Switzerland; 8 Department of Thoracic Surgery, University Hospital Zurich, Zurich, Switzerland; 9 Department of Neuroradiology, Clinical Neuroscience Center, University Hospital Zurich, Zurich, Switzerland

**Keywords:** Lung cancer, Brain metastases, 18F-FDG, PET/CT, OSEM, BSREM

## Abstract

The aim of the study was to analyze the use of block sequential regularized expectation maximization (BSREM) with different *β*-values for the detection of brain metastases in digital fluorine-18 labeled 2-deoxy-2-fluoro-D-glucose (18F-FDG) PET/CT in lung cancer patients. We retrospectively analyzed staging/restaging 18F-FDG PET/CT scans of 40 consecutive lung cancer patients with new brain metastases, confirmed by MRI. PET images were reconstructed using BSREM (*β*-values of 100, 200, 300, 400, 500, 600, 700) and OSEM. Two independent blinded readers (R1 and R2) evaluated each reconstruction using a 4-point scale for general image quality, noise, and lesion detectability. SUVmax of metastases, brain background, target-to-background ratio (TBR), and contrast recovery (CR) ratio were recorded for each reconstruction. Among all reconstruction techniques, differences in qualitative parameters were analyzed using non-parametric Friedman test, while differences in quantitative parameters were compared using analysis of variances for repeated measures. Cohen’s kappa (*k*) was used to measure inter-reader agreement. The overall detectability of brain metastases was highest for BSREM200 (R1: 2.83 ± 1.17; R2: 2.68 ± 1.32) and BSREM300 (R1: 2.78 ± 1.23; R2: 2.68 ± 1.36), followed by BSREM100, which had lower accuracy owing to noise. The highest median TBR was found for BSREM100 (R1: 2.19 ± 1.05; R2: 2.42 ± 1.08), followed by BSREM200 and BSREM300. Image quality ratings were significantly different among reconstructions (*p* < 0.001). The median quality score was higher for BSREM100-300, and both noise and metastases’ SUVmax decreased with increasing *β*-value. Inter-reader agreement was particularly high for the detectability of photopenic metastases and blurring (all *k* > 0.65). BSREM200 and BSREM300 yielded the best results for the detection of brain metastases, surpassing both BSREM400 and OSEM, typically used in clinical practice.

**Supplementary Information:**

The online version contains supplementary material available at 10.1007/s10278-021-00570-y.

## Introduction

Brain metastases (BM) are the most frequent intracranial tumors in adults. Lung cancer, breast cancer, melanoma, renal cell carcinoma (RCC), and colorectal cancer (CRC) are the most common solid tumors associated with BM. BM are associated with higher morbidity and mortality, independently of the type of primary tumor, with an overall survival of less than 2 years [1].

BM occur in approximately 10–20% of lung cancer patients with metastatic disease and approximately 30–50% of patients with non-small-cell lung cancer (NSCLC) will eventually develop BM [2–4]. BM are diagnosed more frequently in small-cell lung cancer (SCLC) compared to other types of lung cancer and are present at initial staging already in approximately 10% of patients [5, 6]. In NSCLC patients, adenocarcinoma subtype, advanced nodal status, advanced tumor stage, and young patient age are known risk factors for the metachronous development of BM [7–9]. The reported overall survival at 60 months is 68% in stage IB, while it is less than 10% in stage IVB [4].

Gadolinium-enhanced magnetic resonance imaging (MRI) is the gold standard for diagnosing BM non-invasively. Brain MRI is not recommended by guidelines for screening in the majority of solid tumors in the absence of suspicious clinical symptoms. In lung cancer, brain MRI is generally recommended for NSCLC stage III and IV [10, 11]. For lower NSCLC stages, recommendations in guidelines vary: while the ESMO guidelines acknowledge usefulness of MRI in stages I and II [12], MRI is considered optional in stage IB by the NCCN guidelines [13] and is encouraged in all stages if there is curative intent by the NICE guidelines [14]. In SCLC, brain MRI is generally recommended in all stages [13, 15].

The staging of lung cancer is among the most widely acknowledged indications for 2-deoxy-2-[18F]fluoro-D-glucose (18F-FDG) positron-emission tomography (PET)/computed tomography (CT) worldwide. It is considered particularly helpful for the detection of lymph node metastases and distant metastases [16]. In particular, 18F-FDG-PET/CT is recommended for staging NSCLC due to its excellent ability (sensitivity 93%, specificity 96%) to detect adrenal and bone metastases, if baseline CT is negative (level of evidence A). 18F-FDG-PET/CT is also proposed in oligometastatic NSCLC patients potentially eligible for treatment. In SCLC patients, although data are still not sufficient (level of evidence C), 18F-FDG-PET/CT is also proposed for staging, particularly for the detection of bone metastases [17–19]. However, the detection rate of BM by 18F-FDG PET/CT is consistently low, ranging between 1 and 2.1% [20–22]. This is owing to the low spatial resolution of PET on the one hand, and the high physiologic FDG avidity of brain background on the other hand, which leads to a low target-to-background ratio (TBR) with poor contrast and poor delineation of cerebral lesions [23–26]. Nevertheless, 18F-FDG PET/CT scans may lead to an incidental detection of BM in patients in the absence of brain MRI, both in early NSCLC stages and in patients at restaging.

Novel iterative Bayesian penalized likelihood reconstruction algorithms, such as block sequential regularized expectation maximization (BSREM), have improved the detectability of small-sized, faintly FDG-avid lesions with low TBR, which holds true for a proportion of brain metastases [27–29]. With BSREM, the optimal reconstruction results depend on the global strength of the ﻿regularization term (*β*-value) [30–33]. For whole-body 18F-FDG scans in oncology, the *β*-value is typically set to 400–450 [34–37]. This *β*-value range represents the clinical standard also at our institution. However, some preliminary data highlight that brain 18F-FDG image quality might benefit from different *β*-values in BSREM reconstruction [38].

The aim of our study was to compare block sequential regularized expectation maximization (BSREM) with different *β*-values and ordered subset expectation maximization (OSEM) algorithms, in order to define which reconstruction algorithm is most appropriate for brain metastases detection in digital 18F-FDG PET/CT.

## Material and Methods

### Patient Selection

We retrospectively analyzed a cohort of 492 consecutive patients, who underwent a clinically indicated 18F-PET/CT scan on a digital scanner for the staging/restaging of lung cancer at the University Hospital of Zürich between May 2017 and January 2020.

The primary inclusion criteria for this retrospective observational study were (a) patient consent for the use of medical data for retrospective studies, (b) histologically proven lung cancer, (c) 18F-FDG PET/CT scan acquired on a digital scanner with silicon photomultiplier (SiPM) technology, and (d) new onset of brain metastases confirmed by gadolinium-enhanced MRI, performed within 30 days of PET/CT. Patients were excluded if (a) age < 18 years old, (b) simultaneous presence of other clinically manifest tumor entity, and (c) no PET raw data available. Finally, out of the 492 patients, a total of 40 patients with MRI-confirmed brain metastases were included (refer to flow chart in supplemental fig. S1).

Our study was approved by the local ethics committee and was conducted in compliance with ICH-GCP rules and the Declaration of Helsinki.

### PET/CT Acquisition

Patients fasted for at least 4 h prior to the scan, and blood glucose levels were below 160 mg/dl at the time of 18F-FDG injection. All patients underwent a PET/CT scan on a digital scanner with silicon photomultiplier (SiPM) technology (GE Discovery Molecular Insights—DMI PET/CT, GE Healthcare, Waukesha, WI). The injected tracer activity was 202.00 ± 73.84 MBq of 18F-FDG. After an uptake time of 60 min and following CT acquisition both for attenuation correction and anatomical correlation (from the vertex of the skull to the mid-thighs or to the feet), PET data were acquired in 3-dimensional time-of-flight (TOF) mode, covering the identical anatomical region of the CT, with 2.5 min/bed position and 6–11 bed positions per patient (23% overlap), depending on patient size.

### Bayesian Penalized Likelihood Reconstruction Algorithms

Ordered subset expectation maximization (OSEM) PET image reconstruction was introduced in 1994 and is still a widely used reconstruction algorithm for PET images. OSEM divides the image data into subsets, to whom an expectation maximization is applied, leading to less artifacts and image noise compared to older reconstruction algorithms [30, 31]. More recently, point spread function modeling was implemented with OSEM (OSEM_PSF_), which further improved the signal-to-noise ratio (SNR) [39, 40]. OSEM images with 25 iterations provide almost perfect SUV quantitation, but at the expense of severe image noise. Hence, OSEM is typically stopped after 2–4 iterations with subsequent inaccuracies in quantitative assessment [41–43].

Recently, the switch from large conventional photomultiplier tubes to small solid state silicon-based photomultipliers (SiPMs) lead to the improvement of several technical aspects: e.g., higher spatial resolution (3–4 mm pixels for digital versus 4–6 mm pixels for conventional system), higher geometric sensitivity (by removing collimation and using a longer axial FOV), and a high effective sensitivity owing to time-of-flight (TOF) measurements (from 500–600 to 200–300 ps) [44–47].

PET image reconstruction has so evolved further with the advent of Bayesian penalized likelihood (BPL) reconstruction algorithms, such as block sequential regularized expectation maximization (BSREM — Q.Clear; GE Healthcare). BPL reconstruction algorithms increase the accuracy of lesion quantitation compared to OSEM by maximizing signal–to-noise ratio (SNR), while achieving almost full convergence [48–50], yielding advantages in oncological populations [32, 51–53]. With BSREM, the optimal reconstruction results depend on the global strength of the ﻿regularization term (*β*-value), which again depends on several aspects (e.g., dosage, noise, radionuclide used, anatomical area of examination) [30–33, 54].

### Image Reconstruction

In all 40 patients with MRI-confirmed brain metastases, dedicated brain PET image datasets were reconstructed with different standardized settings (all with a 256 × 256 pixel matrix):

1. OSEM: 3 iterations, 16 subsets, FWHM of 6.3 mm, 1:4 *Z*-axis filter and 6.4 mm Gaussian filter with both time-of-flight (TOF) and point spread function (PSF) modeling (OSEM_PSF_; VUE Point FX with SharpIR, GE Healthcare).
2. BSREM (Q.Clear, GE Healthcare) with both TOF and PSF and *β*-values of 100 (BSREM_100_), 200 (BSREM_200_), 300 (BSREM_300_), 400 (BSREM_400_), 500 (BSREM_500_), 600 (BSREM_600_), 700 (BSREM_700_).

### Qualitative Imaging Analysis

A total of 320 reconstructed PET/CT datasets (40 patient studies, each with the eight aforementioned different reconstructions) were evaluated by two readers (D.P. and V.L, with 7 and 6 years of experience in nuclear medicine, respectively) blinded to the reconstruction method used. All scans were reviewed independently on a dedicated workstation (Advantage Workstation, Version 4.6; GE Healthcare) and in random order. Readers identified cerebral metastases by reading both PET and CT images. Readers were blinded to all clinical information, except the presence of brain metastases from a primary lung tumor. In case of discrepancy of image rating, a final decision was made by consensus including a third reader (MWH, with 11 years of experience in nuclear medicine and radiology).

Readers first rated the general image quality; for this purpose, datasets were viewed using axial views with reformatted sections. The two readers evaluated several qualitative aspects using a 4-point grading scale: (a) general image quality (GIq) score of each reconstructed image, based on the ability to discriminate grey matter (GM) from white matter (WM); (b) noise score; and (c) overall lesion detectability. Both readers assessed overall brain GIq and noise score as well as neocortex, basal ganglia, cerebellum and brainstem GIq, and noise, respectively. These three 4-point grading scales are summarized in Table 1.

**Table 1:** Image grading scores

**Category**	**General image quality (based on GM/WM score)**	**Noise score**	**Lesion detectability score**
1	Poor (inadequate image with blurring)	Slight (almost none)	Poor
2	Fair (diagnostically relevant image blurring)	Fair (diagnostic irrelevant)	Average
3	Good (diagnostic irrelevant image blurring)	Moderate (diagnostic relevant)	Good
4	Excellent (almost no blurring)	Severe (marked)	Very good

*GM* grey matter, *WM* white matter

Based on PET images, five other dichotomic (Y/N) qualitative scores were also assessed: presence of hypermetabolic metastases, hypometabolic metastases (defined as hypometabolic compared to brain cortex and basal ganglia), edema, mass effect, and blurring of the target lesion.

### Quantitative Imaging Analysis

Quantitative analyses were performed by the same two independent blinded readers (D. P. and V. L). The maximum standardized uptake value (SUVmax) of each brain metastasis was recorded using a standard volume of interest (VOI) tool on PET/CT images. Herewith, the VOI was automatically propagated to cover exactly the same volume in all eight different reconstruction datasets to ensure consistency of the area selected among different reconstructions in order to extract semiquantitative parameters. Moreover, background mean standardized uptake value (SUVmean) was assessed segmented manually using a banana-shaped VOI in normally appearing contralateral frontal and parietal brain parenchyma, including WM and GM. Based on these measurements, a target-to-background ratio (TBR) was calculated for each brain metastasis, defined as metastasis’ SUVmax/background SUVmean.

We also defined a contrast recovery (CR) ratio comparing the target-to-background ratio of a BSREM reconstruction (numerator) to the target-to-background ratio of the reference OSEM reconstruction (denominator):

$${CR}=\frac{\left(\frac{SUVmax}{SUVmean_{bkgnd}}\right)-1}{\left(\frac{SUVmax_{ref}}{{SUVmean_{bkgnd_{ref}}}}\right)-1}$$

where SUVmax is obtained from the brain metastasis and SUVmean from the background (“bkgnd”). Finally, “ref” indicates the reference reconstruction, as previously explained by ter Voert er al. [31]. The CR ratio is intended to highlight the advantage or disadvantage of each reconstruction compared to the ones used in clinical routine. In our study, we used both OSEM and BSREM_400_ as reference reconstructions.

### Statistical Analyses

Categorical variables are expressed as proportions, and continuous variables are presented as mean ± standard deviation (SD) or median (range), depending on the distribution of values. For qualitative parameters, we compared the eight reconstruction techniques with respect to the qualitative image ratings (GIq, noise score, overall lesions detectability) using the non-parametric Friedman test for multiple samples. For quantitative parameters, brain metastases’ SUVmax, background SUVmean, TBR, and CR were compared among all reconstruction techniques, using analysis of variances for repeated measures, with post-hoc Bonferroni corrections to adjust for multiple comparisons.

Further, differences in brain metastases’ SUVmax were compared between patients with—low (< 25, *n* = 21) and high (> 25, *n* = 19) BMI,—low (≤ 2.0 MBq/kg body weight; *n* = 12) and a high (> 2.0 MBq/kg body weight; *n* = 28) administered 18F-FDG activity,—low (< 5.5 mmol/l; *n* = 18) and high (> 5.5 mmol/l; *n* = 22) glucose levels, and—small (< 1.5 cm in longest diameter; *n* = 29) and large (> 1.5 cm in longest diameter; *n* = 11) metastases using Mann–Whitney *U* test.

Cohen’s kappa coefficient (*k*) was used to measure interrater agreement for qualitative scores, such as GIq, noise score, overall lesions detectability and the presence of hypermetabolic metastases, hypometabolic metastases, edema, mass effect, and blurring of the target lesion. Cohen’s kappa was interpreted as follows: ≤ 0 no agreement, 0.01–0.20 as none to slight, 0.21–0.40 fair, 0.41– 0.60 moderate, 0.61–0.80 substantial, and 0.81–1.00 almost perfect agreement [55]. Statistical significance was considered for *p* < 0.05. Statistical analyses were performed using IBM SPSS version 26.0 (IBM, Armonk, NY, USA) [56].

## Results

Of the 40 patients included in this study (25 M, 15F), 77.5% underwent PET/CT for staging and 22.5% for restaging. The majority of patients were already deemed stage IV (70%), based on CT imaging performed before the PET/CT scan (thereof 8/28 M1a, 6/28 M1b, 14/28 M1c). Of the 40 patients, 5/40 (12.5%) had SCLC and 35/40 (87.5%) had NSCLC (4/40 (10.0%) squamous cell carcinoma, 24/40 (60.0%) adenocarcinoma, and 7/40 (17.5%) poorly differentiated carcinoma).

Based on the reference reconstructions for digital PET/CT scan (BSREM_400_), *clinical* PET/CT reports (not further analyzed as part of this study) for brain metastases were positive, doubtful and negative in 16/40 (40%), 2/40 (5%), and 22 (55%) patients, respectively.

The overall number of BM detected retrospectively with PET/CT in our study were 67, 69, 70, 65, 59, 53, 48, and 49 at BSREM_100_, BSREM_200_, BSREM_300_, BSREM_400_, BSREM_500_, BSREM_600_, BSREM_700_, and OSEM, respectively. The mean number of BM detected was 1.63 ± 1.48 (median = 1; 0–7) per patient at *clinical* 18F-FDG PET/CT with BSREM_400_ reconstruction versus 4.42 ± 5.93 (median = 2; 1–30; total 177) at MRI. BSREM_300_ reconstruction showed the highest mean number of BM detected per patient (1.75 ± 1.46; median = 1; 1–7). Patient and tumor characteristics are listed in Table 2.

**Table 2:** Demographic data of study subjects (*n* = 40)

**Patient and tumor characteristics**	
*PET/CT scan*, *n* (%) Staging Restaging	35 (87.5%) 5 (12.5%)
*Gender*, *n* (%) Male Female	25 (62.5%) 15 (37.5%)
*Age* (years), median (range)	66.5 (31–89)
*Activity injected* (MBq), median (range)	230.0 (89–302)
*Uptake time* (min), median (range)	57.5 (47–78)
*Blood glucose level* (mmol/L), median (range)	5.6 (4.5–7.8)
*Weight* (kg), median (range)	75.5 (47–111)
*Height* (cm), median (range)	172.0 (151–185)
*BMI* (*kg/m*^*2*^), median (range)	24.7 (18.4–37.5)
**Primary lung characteristics**	
*Histological type*, *n* (%) * Small-cell lung carcinoma (SCLC)* * Non-small-cell lung carcinoma (NSCLC)* * Squamous cell carcinoma* * Adenocarcinoma* * Poorl differentiated*	5 (12.5%) 35 (87.5%) 4 (10.0%) 24 (60.0%) 7 (17.5%)
*Location*, *n* (%) * Right lung* * Left lung* * Unknown*	22 (55.0%) 17 (42.5%) 1 (2.5%)
*Stage before *^*18*^*F-FDG PET/CT*, *n* (%) * I* * IIA* * IIIB* * IV* * Unknown*	1 (2.5) 1 (2.5) 1 (2.5) 28 (70.0) 9 (22.5)
**Brain metastasis characteristics**	
Clinical PET/CT report on brain metastases detection*, *n* (%) * Positive* * Doubtful* * Negative*	16 (40.0%) 2 (5.0%) 22 (55.0%)
*Number of brain metastases per patient detected with *^*18*^F-FDG PET/CT** mean ± DS (median, range) * BSREM*_*100*_ * BSREM*_*200*_*BSREM*_*300*_ * BSREM*_*400*_ * BSREM*_*500*_ * BSREM*_*600*_ * BSREM*_*700*_ * OSEM*	1.68 ± 1.38 (1; 1–7) 1.73 ± 1.43 (1; 1–7) 1.75 ± 1.46 (1; 1–7) 1.63 ± 1.48 (1; 0–7) 1.48 ± 1.50 (1; 0–7) 1.33 ± 1.40 (1; 0–7) 1.20 ± 1.28 (1; 0–6) 1.23 ± 1.27 (1; 0–6)

^*^Based on findings detected on *clinical* PET with BSREM reconstruction with a *β* value of 400, which represents the clinical standard at our institution[33–36]

^**^Based on inter-reader agreement: readers identified cerebral metastases by reading both PET and CT images. In case of discrepancy of image rating, a final decision was made by consensus including a third reader, with 11 years of experience in nuclear medicine and radiology

### Qualitative Image Results

The results of the subjective image assessment regarding general image quality (GIq) score, the noise score, and the overall lesion detectability are given in Table 3. The results regarding the other five dichotomic qualitative scores (hypermetabolic metastases, hypometabolic metastases, edema, mass effect, and blurring of the target lesion) and the ones with regard to GIq and noise score of the four analyzed brain structures (neocortex, basal ganglia, cerebellum, and brainstem) are reported in supplemental table S1 and supplemental table S2, respectively.

**Table 3:** Results of subjective PET image quality rating and PET parameters for different reconstruction algorithms

**Reconstruction**	**General image quality score**				**Noise score**				**Lesion detectability score**			
	**Reader 1**		**Reader 2**		**Reader 1**		**Reader 2**		**Reader 1**		**Reader 2**	
	**Mean**	**SD**	**Mean**	**SD**	**Mean**	**SD**	**Mean**	**SD**	**Mean**	**SD**	**Mean**	**SD**
**BSREM**_**100**_	2.14	1.13	3.63	0.59	3.73	0.67	3.9	0.49	2.60	1.08	2.60	1.37
**BSREM**_**200**_	2.59	1.10	3.68	0.50	3.03	0.76	3.4	0.83	2.83	1.17	2.68	1.32
**BSREM**_**300**_	2.58	1.04	3.15	0.72	2.03	0.88	2.75	0.94	2.78	1.23	2.68	1.36
**BSREM**_**400**_	2.39	1.02	2.90	0.64	1.44	0.72	2.00	1.07	2.7	1.28	2.60	1.35
**BSREM**_**500**_	2.24	1.01	2.46	0.77	1.13	0.42	1.40	0.80	2.56	1.25	2.35	1.38
**BSREM**_**600**_	1.96	0.83	2.13	0.76	1.06	0.31	1.02	0.15	2.30	1.2	2.28	1.35
**BSREM**_**700**_	1.63	0.70	1.91	0.71	1.01	0.08	1.02	0.15	2.08	1.16	2.25	1.31
**OSEM**	1.58	0.74	1.94	0.84	1.09	0.41	1.23	0.72	2.30	1.11	2.28	1.33
**Reconstruction**	**SUVmax**				**SUVmean**				**TBR**			
	**Reader 1**		**Reader 2**		**Reader 2**		**Reader 2**		**Reader 1**		**Reader 2**	
	**Mean**	**SD**	**Mean**	**SD**	**Mean**	**SD**	**Mean**	**SD**	**Mean**	**SD**	**Mean**	**SD**
**BSREM**_**100**_	13.47	6.69	13.85	6.62	6.44	2.21	5.85	1.63	2.19	1.05	2.42	1.08
**BSREM**_**200**_	11.68	5.95	11.91	6.02	6.11	1.96	5.81	1.60	1.96	0.93	2.08	0.95
**BSREM**_**300**_	10.75	5.37	10.95	5.48	6.03	1.91	5.81	1.60	1.82	0.83	1.91	0.85
**BSREM**_**400**_	10.15	5.08	10.32	5.02	5.8	1.71	5.81	1.60	1.76	0.76	1.79	0.77
**BSREM**_**500**_	10.01	4.83	9.87	4.63	5.77	1.70	5.90	1.65	1.75	0.72	1.68	0.67
**BSREM**_**600**_	9.95	4.45	9.53	4.31	5.74	1.63	5.97	1.72	1.75	0.69	1.61	0.62
**BSREM**_**700**_	9.84	4.21	9.26	4.04	5.78	1.60	5.94	1.70	1.72	0.67	1.58	0.61
**OSEM**	9.34	3.44	9.11	3.6	5.67	1.80	5.85	1.58	1.68	0.59	1.57	0.57

*BSREM* block sequential regularized maximization, *OSEM* ordered subset expectation maximization, *TBR* target-to-background ratio

General image quality, noise score, and overall lesions detectability were rated significantly different among all reconstruction algorithms at Friedman test for multiple samples ﻿(*p* < 0.001 for both readers).

The median quality score was by trend higher for the BSREM reconstruction with lower *β*-values, with a similar decreasing trend of qualitative score rating for both readers. In particular, the median quality score for BSREM_100_ was 2.14 ± 1.13 (reader 1) and 3.63 ± 0.59 (reader 2), for BSREM_200_ 2.59 ± 1.10 (reader 1) and 3.68 ± 0.50 (reader 2), and for BSREM_300_ 2.58 ± 1.04 (reader 1) and 3.68 ± 0.50 (reader 2). The most evident inter-reader discrepancy was due to a higher average evaluation by reader 2 compared to reader 1, which is accentuated for the BSREM_100_ reconstruction (Table 3). This data is reflected in the low agreement of the two readers for this particular score with a weighted *k* < 0.05 for the majority of reconstructions, with the exception of BSREM_200_ (*k* = 0.268 and 95% confidence interval (CI) 0.09–0.44) and OSEM (*k* = 0.306 and 95% CI 0.12–0.48) (supplemental table S3).

**Fig. 1:**
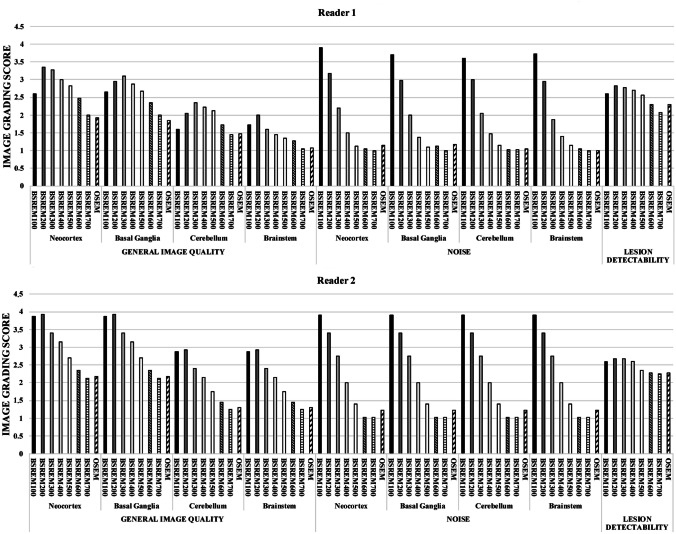
Visual scoring of general image quality and noise of neocortex, basal ganglia, cerebellum, and brainstem, respectively, and overall lesion detectability evaluated by the two readers per arbitrary image grading score, as described in Table 2

In contrast, inter-reader agreement was high for both noise and lesion detectability, both decreasing with higher *β*-value and OSEM. OSEM reconstruction presented a similar performance, as BSREM_700_. The overall detectability of brain metastases was higher for BSREM_200_ and BSREM_300_ for both readers compared to all other reconstructions. Owing to deterioration of image quality by noise, BSREM_100_ accuracy was lower. In the analysis of brain structures, both readers assigned a lower GIq to cerebellum and brainstem compared to neocortex and basal ganglia with a similar trend, even if GIq score by reader 2 was generally higher compared to reader 1, as shown in Fig. 1.

Finally, with regard to the dichotomic scores, agreements were surprisingly high for photopenic brain metastases, which turned out to be highly detectable regardless of the reconstruction algorithm used (supplemental Fig. 2), and for metastases’ blurring, which was slightly higher with higher *β*-value and OSEM for reader 2.

**Fig. 2:**
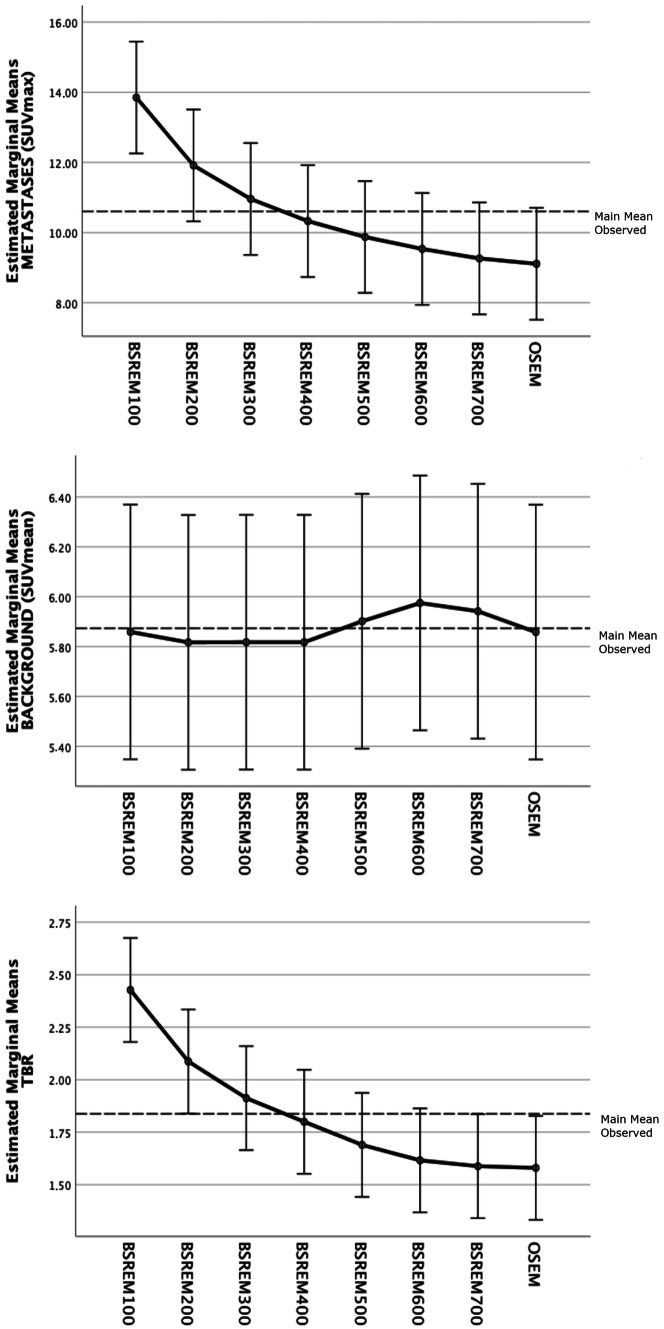
Profile plots of the estimated marginal means of PET parameters (brain metastases’ SUVmax, background SUVmean, and TBR) derived by pairwise comparisons of different reconstruction algorithms

### Quantitative Image Results

Mean values of PET parameters for both readers are given in Table 3; mean differences of PET parameters and *p* values of pairwise comparisons using different reconstruction algorithms are given in supplemental table S4.

Apart from pairwise comparisons (supplemental table S4), only BSREM_100_ was significantly different from BSREM_500_ (*p* = 0.017), BSREM_600_ (*p* = 0.006), BSREM_700_ (*p* = 0.002), and OSEM (*p* = 0.002) for metastases’ SUVmax and from BSREM_400_ (*p* = 0.014), BSREM_500_ (*p* = 0.001), BSREM_600_ (*p* = 0.0001), BSREM_700_ (*p* = 0.0001), and OSEM (*p* = 0.0001) for TBR. The estimated marginal means presented in Fig. 2 reveal a significant and progressive reduction of both PET parameters with increasing *β*-value, with very similar results for BSREM_700_ and OSEM.

**Fig. 3:**
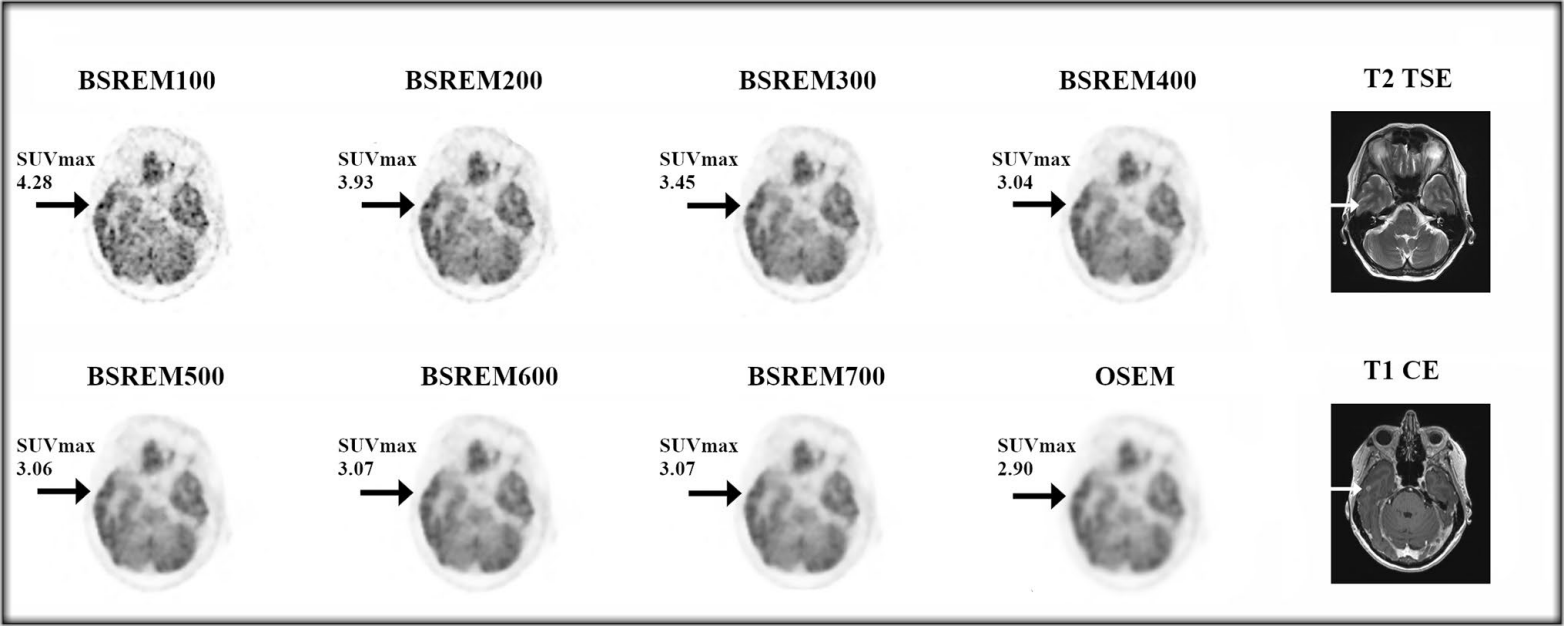
Exemplary case of a 62-year-old patient undergoing restaging ^18^F-FDG PET/CT performed for an adenocarcinoma of the left lung with lymph node metastases and brain metastases (T1c Nx M1b, stage IVB). The MR images performed 20 days after PET/CT shows a tiny brain metastasis in the right-sided temporal lobe (white arrows), which was detected by both readers only on BSREM_100_ and BSREM_200_ reconstructions and is faintly present also on BSREM_300_

These data are corroborated also by the results of the contrast recovery (CR) ratio comparing PET parameters of a given reconstruction with the reference reconstruction (supplemental fig. S3). The box plot representation of the CR ratio with BSREM_400_ as reference reconstruction reveals an added value of BSREM_100_, BSREM_200_, and BSREM_300_ in comparison to the other reconstruction algorithms, leading to a better definition of tiny lesions in BSREM with lower *β*-values, as shown in Fig. 3.

Finally, the Mann–Whitney *U* test for differences in brain metastases’ SUVmax showed no significant impact of lesion size on SUVmax, while BMI > 25 and MBq/kg < 2.0 impacted SUVmax for BSREM reconstruction with higher *β*-value and for OSEM, but without significance, except for BMI > 25 in OSEM (supplemental table S5).

## Discussion

To the best of our knowledge, this study is the first one that sought to analyze different PET reconstruction algorithms for the assessment of brain metastases in patients with lung cancer, using a latest-generation silicon-based digital PET/CT scanner.

The major findings of our study are as follows: (1) BSREM with lower *β*-values (namely, BSREM_100_, BSREM_200_, and BSREM_300_) is better suited for the detection of BM than BSREM with higher *β*-values or OSEM; (2) BSREM reconstruction leads to a significant increase of SUVmax and TBR, being most prominent with lower *β*-values; (3) despite that, BSREM_100_ sensitivity is affected by the presence of high noise levels compared to other reconstructions; (4) PET/CT sensitivity for BM detection is not affected by brain lesion size, regardless of the reconstruction algorithm used; (5) BMI > 25 and MBq/kg < 2.0 could reduce the sensitivity of both high *β*-value BSREM and OSEM for BM detection (but future prospective data are needed to validate this finding), and (6) both readers agreed that BSREM_200-300_ leads to a significant increase in BM detectability compared to BSREM_400_, which is normally used in clinical practice for whole-body exams.

Several studies have previously compared different PET reconstruction algorithms with regard to image quality and quantitative parameters in whole-body oncological subsets, but none of them focused on 18F-FDG brain PET/CT images in oncological subsets. Notably, all studies analyzing the impact of different reconstructions in 18F-FDG PET/CT whole-body exams [27, 34–36, 57] reported an ideal *β*-value between 400 and 500 for BSREM reconstructions. Two other studies focusing on the evaluation of prostate cancer with 68 Ga-PSMA-11 [31, 58] reported higher ideal *β*-values for this high energy positron emitter, slightly higher with ter Voert et al. (PET/MR study, BSREM_500-600_) and considerably higher with Lindström et al. (PET/CT study, BSREM_900_), which, however, might be explained by the known differences in *β*-value characteristics between PET/MR and PET/CT. In another study, Lindström et al. [59] identified tracer-specific ranges of *β*-values for BSREM reconstruction according to the different biodistribution of the tracers, different background uptake, and different positron range of 18F-FDG, 11C-acetate, and 68 Ga-DOTATOC in tissue. According to their findings, 18F-FDG requires lower *β*-values without compromising the image quality in terms of increased noise, while 68 Ga-DOTATOC images benefit from higher *β*-values.

These data corroborate the impossibility to identify one single optimal *β*-value suitable for all radiopharmaceuticals and all scan indications. To the best of our knowledge, to date only Shkumat et al. [60] have quantified the diagnostic performance of OSEM and BSREM (*β*-values 200, 350, and 500) reconstructions in brain 18F-FDG PET/CT images of twenty-five pediatric epilepsy patients on a digital silicon photomultiplier system, stratified also by different acquisition times (45 s, 90 s, 180 s, 300 s) in order to simulate reduced count density. They report that pediatric brain 18F-FDG PET/CT images remain diagnostic with a reduction of count density by 40% when using a *β*-value of 350–500. Moreover, they also identified a reduction of image quality of cortex, basal ganglia, and thalamus when applying lower *β*-values due to increasing noise, particularly in the thalamus. In their study, the highest spatial resolution was reported for a *β*-value of 200–300.

Results of our study are in line with these findings, with BSREM_200_ and BSREM_300_ leading to a better general image quality being counterbalanced by acceptable background noise, in contrast to BSREM_100_. In addition, based on the subjective evaluation of the two readers, spatial resolution seemed to be higher when applying BSREM100-300, whereas higher *β*-values resulted in a loss of gray matter/white matter contrast, especially for smaller structures, such as basal ganglia and brainstem. A quantitative assessment of spatial resolution should be performed to validate this hypothesis.

These findings may not only be of significance in the oncological field for BM detection, but also in neurodegenerative disorders. Recently, Lindström et al. [38] have evaluated how different *β*-values (BSREM; TOF, PSF, *β*-value 75–300) can affect quantitative measures and software-aided assessment of pathologies in patients with neurodegenerative diseases compared to cognitively normal controls, in both 18F-flutemetamol and 18F-FDG PET/CT imaging. They conclude that BSREM image reconstructions should be used with caution when a normal’s database was collected based on images acquired with OSEM reconstruction. Moreover, they reported that TOF, PSF, and BSREM either increased or decreased the relative uptake difference to the normal’s database within the software, depending on the radiotracer and chosen reference area.

While the right choice between BSREM_200_ and BSREM_300_ remains arbitrary and somewhat subjective, it seems clearer that intermediate-high *β*-value (600–700) are not suitable for a correct evaluation of brain 18F-FDG PET/CT images, both qualitatively and quantitatively with regard to PET parameters. BSREM_600_ and BSREM_700_ show a considerable reduction in the number of detected metastases (mean 1.33 ± 1.40 and 1.20 ± 1.28, respectively), compared to BSREM_300_ (mean 1.75 ± 1.46). This comes along with a significant drop of the measured semi-quantitative PET parameter values with BSREM_600_ and BSREM_700_, which were within the range of those measured in OSEM. These data are in line with previous findings by Caribé et al. [61], who already reported that BSREM_750_ has a resolution comparable to OSEM, but with a background noise level reduced by a factor of 4 (coefficient of variation, COV), overall reducing the detectability of small lesions in BSREM_750_ compared to lower *β*-value BSREM, as shown in the example in supplemental fig. S4.

Literature data suggests a correlation between lesion dimension and PET/CT detectability [51], which was, however, not observed in our study. One explanation might be the heterogeneity of biological behavior of BM, leading to varying FDG patterns (hypermetabolic, isometabolic, and hypometabolic, the latter indicating prominent perifocal edema), as already mentioned by Hjorthaug et al. [21]. Interestingly, our results suggest a possible association of BMI > 25 and MBq/kg < 2.0 with lower sensitivity of both BSREM with higher *β*-values and OSEM, although without statistical significance. Recently, Messerli et al. [32] highlighted how the optimal *β*-value choice in whole-body 18F-FDG PET/CT could depend on the administered activity (MBq/kg < or > 2.0). Future prospective data with a larger cohort of patients are needed to validate these findings.

To conclude, despite all the known limitations of 18F-FDG PET/CT images in this field, the use of BSREM reconstruction with *β*-values of 200–300 could increase the sensitivity of 18F-FDG PET/CT images to detect brain metastases.

We acknowledge that MR imaging undoubtedly represents the reference standard for the cerebral staging of advanced lung cancer patients. As mentioned, a staging MRI is considered mandatory for NSCLC patients stage II or higher [62–64]. However, data from more than 457,000 NSCLC patients reported by Wagar et al. [65] proofs that — while the prevalence of BM at initial presentation doubles from stage I to stage II (from 2.3 to 4.6%) — only 74% of stage II patients actually undergo MR imaging (27% of stage I patients). Even in stages II and IV (BM prevalence 10.8% and 12.1%, respectively), a brain MRI was available only in 92% and 88% of patients, respectively.

Furthermore, if no BM where detected at initial staging, and in the absence of neurological symptoms, a regular MRI surveillance of the brain is oftentimes not performed, also in subjects undergoing regular follow-up PET/CT scans for whole-body surveillance. In these subjects, adequate PET imaging of the brain — as part of the whole-body exam — might be critical. Notably, lung cancer restaging represents one of the most common PET indications, owing to the general high incidence of lung cancer and owing to available reimbursement for this disease in most countries with PET reimbursement schemes. Also, some patients cannot undergo MRI examinations owing to contraindications. In these subjects, adequate PET imaging of the brain might assume a more prominent role, possibly in conjunction with contrast-enhanced (ce)-CT, which was however not analyzed in our study.

Hence, the detection of BM by 18F-FDG PET/CT might have a positive impact on clinical management and patient outcome not only in advanced lung cancer patients, but also in all other solid tumors often metastatic to the brain (breast cancer, melanoma, RCC, and CRC), and in which brain MRI is prescribed only in case of high suspicion and/or presence of neurological symptoms.

Our study is not exempt from limitations. First, although readers were blinded to the type of reconstruction used, an experienced reader may recognize the actual algorithm used based on the reconstructed images. Second, despite BSREM_200_ and BSREM_300_ yielded best results for the detection of brain metastases, a statistically significant difference in the number of brain metastases detected among the different reconstruction types was not found. The number of patients in this single center study is comparably small, and therefore, conclusions drawn from the present analysis await further proof in larger (and ideally multicentric) observations. Future studies are also warranted to assess the impact of BSREM depending on different primary cancer origin, different metabolic behavior of BM, and different brain regions involved for a better stratification of diagnostic accuracy, clinical management, and patient outcome. Since a detailed analysis of OSEM was not the thrust of our study, OSEM was not optimized, but clinically standard OSEM served as a reference only.

## Conclusion

BSREM_200_ and BSREM_300_ yielded better results for the detection of brain metastases, being superior to BSREM_400_ and OSEM, which are normally used in clinical practice for whole-body exams. With BSREM_200_ and BSREM_300_, higher SUVmax, TBR, and an acceptable background noise translate into higher image quality and tumor conspicuity. In clinical routine, it might be useful to separately reconstruct and analyze such brain PET images in whole-body exams for the staging and restaging of lung cancer patients, particularly in the absence of synchronous brain MR imaging.

## Supplementary Information

Below is the link to the electronic supplementary material.

Supplementary file1 (DOCX 889 KB)

Supplementary file2 (JPG 37 KB)

Supplementary file3 (PNG 367 KB)

Supplementary file4 (PNG 491 KB)

Supplementary file5 (PNG 819 KB)

## Data Availability

The datasets used and/or analyzed during the current study are available from the corresponding author on reasonable request.

## Abbreviations

18F-FDG: Fluorine-18 labeled 2-deoxy-2-fluoro-D-glucose
AJCC: American Joint Committee on Cancer
BMI: Body mass index
BPL: Bayesian penalized likelihood
BSREM: Block sequential regularized expectation maximization
CE: Contrast-enhanced
CECT: Contrast-enhanced computed tomography
CR: Contrast recovery
CT: Computed tomography
MIP: Maximum intensity projection
MRI: Magnetic resonance imaging
MVXD: MultiVane-XD sequence
OSEM: Ordered subset expectation maximization
PET: Positron emission tomography
PSF: Point spread function
SiPM: Silicon photomultiplier
SUVmax: Maximum standardized uptake value
TBR: Target-to-background ratio
TFE: Turbo field echo
TOF: Time of flight
TSE: Turbo spin-echo
VOI: Volume of interest
