# Supplementary material for: BSREM for Brain Metastasis Detection with 18F-FDG-PET/CT in Lung Cancer Patients
Source: J Digit Imaging. 2022 Feb 25;35(3):581–93. doi: 10.1007/s10278-021-00570-y (PMC9156589; doi:10.1007/s10278-021-00570-y)

**SUPPLEMENTAL MATERIAL - TABLES AND TABLES LEGENDS**

**Table S1.** Brain metastases characteristics based on subjective PET evaluation of reader 1 and reader 2.

| **Reconstruction** | **Hypermetabolic metastases** | | | | | | | **Hypometabolic metastases** | | | | | | | **Edema** | | | | | |
| --- | --- | --- | --- | --- | --- | --- | --- | --- | --- | --- | --- | --- | --- | --- | --- | --- | --- | --- | --- | --- |
|  | **Reader 1** | | | | **Reader 2** | | | **Reader 1** | | | | **Reader 2** | | | **Reader 1** | | | | **Reader 2** | |
|  | **n** | | **%** | | **n** | **%** | | **n** | | **%** | | **n** | | **%** | **n** | | **%** | | **n** | **%** |
| **BSREM_100_** | 17 | | 42.5 | | 17 | 42.5 | | 8 | | 20.0 | | 8 | | 20.0 | 22 | | 55.0 | | 14 | 35.0 |
| **BSREM_200_** | 17 | | 42.5 | | 18 | 45.0 | | 8 | | 20.0 | | 8 | | 20.0 | 22 | | 55.0 | | 14 | 35.0 |
| **BSREM_300_** | 17 | | 42.5 | | 18 | 45.0 | | 8 | | 20.0 | | 8 | | 20.0 | 22 | | 55.0 | | 14 | 35.0 |
| **BSREM_400_** | 23 | | 57.5 | | 17 | 42.5 | | 8 | | 20.0 | | 8 | | 20.0 | 19 | | 47.5 | | 14 | 35.0 |
| **BSREM_500_** | 18 | | 45.0 | | 16 | 40.0 | | 8 | | 20.0 | | 8 | | 20.0 | 14 | | 35.0 | | 14 | 35.0 |
| **BSREM_600_** | 16 | | 40.0 | | 15 | 37.5 | | 8 | | 20.0 | | 9 | | 22.5 | 14 | | 35.0 | | 14 | 35.0 |
| **BSREM_700_** | 16 | | 40.0 | | 15 | 37.5 | | 7 | | 17.5 | | 9 | | 22.5 | 13 | | 32.5 | | 14 | 35.0 |
| **OSEM** | 17 | | 42.5 | | 16 | 40.0 | | 8 | | 20.0 | | 8 | | 20.0 | 13 | | 32.5 | | 14 | 35.0 |
|  | | | | | | | | | | | | | | | | | | | | |
| **Reconstruction** | | **Mass effect** | | | | | | | | | **Blurring** | | | | | | | | | |
|  |  | **Reader 1** | | | | | **Reader 2** | | | | **Reader 1** | | | | | **Reader 2** | | | | |
|  |  | **n** | | **%** | | | **n** | | **%** | | **n** | | **%** | | | **n** | | **%** | | |
| **BSREM_100_** | | 11 | | 27.5 | | | 7 | | 17.5 | | 6 | | 15.0 | | | 7 | | 17.5 | | |
| **BSREM_200_** | | 11 | | 27.5 | | | 7 | | 17.5 | | 8 | | 20.0 | | | 7 | | 17.5 | | |
| **BSREM_300_** | | 11 | | 27.5 | | | 7 | | 17.5 | | 8 | | 20.0 | | | 8 | | 20.0 | | |
| **BSREM_400_** | | 11 | | 27.5 | | | 6 | | 15.0 | | 8 | | 20.0 | | | 8 | | 20.0 | | |
| **BSREM_500_** | | 9 | | 22.5 | | | 6 | | 15.0 | | 7 | | 17.5 | | | 9 | | 22.5 | | |
| **BSREM_600_** | | 10 | | 25.0 | | | 6 | | 15.0 | | 7 | | 17.5 | | | 10 | | 25.0 | | |
| **BSREM_700_** | | 9 | | 22.5 | | | 6 | | 15.0 | | 6 | | 15.0 | | | 10 | | 25.0 | | |
| **OSEM** | | 9 | | 22.5 | | | 6 | | 15.0 | | 6 | | 15.0 | | | 8 | | 20.0 | | |
| ***Note****: BSREM = block sequential regularized maximization. GIq =* general image quality. *OSEM = ordered subset expectation maximization* | | | | | | | | | | | | | | | | | | | | |

**Table S2**. Subjective PET image quality and noise rating for neocortex, basal ganglia, cerebellum and brainstem, for different reconstruction algorithms assessed by reader 1 and reader 2 evaluation.

| **Reconstruction** | **Neocortex GIq** | | | | **Basal ganglia GIq** | | | | **Cerebellum GIq** | | | | **Brainstem GIq** | | | |
| --- | --- | --- | --- | --- | --- | --- | --- | --- | --- | --- | --- | --- | --- | --- | --- | --- |
|  | **Reader 1** | | **Reader 2** | | **Reader 1** | | **Reader 2** | | **Reader 1** | | **Reader 2** | | **Reader 1** | | **Reader 2** | |
|  | **Mean** | **SD** | **Mean** | **SD** | **Mean** | **SD** | **Mean** | **SD** | **Mean** | **SD** | **Mean** | **SD** | **Mean** | **SD** | **Mean** | **SD** |
| **BSREM_100_** | 2.60 | 0.95 | 3.88 | 0.40 | 2.65 | 1.21 | 3.88 | 0.40 | 1.60 | 0.90 | 2.88 | 0.40 | 1.73 | 1.03 | 2.88 | 0.40 |
| **BSREM_200_** | 3.35 | 0.89 | 3.93 | 0.26 | 2.95 | 1.03 | 3.93 | 0.26 | 2.05 | 0.90 | 2.93 | 0.26 | 2.00 | 0.96 | 2.93 | 0.26 |
| **BSREM_300_** | 3.28 | 0.71 | 3.40 | 0.59 | 3.10 | 0.87 | 3.40 | 0.59 | 2.35 | 0.89 | 2.40 | 0.60 | 1.60 | 0.77 | 2.40 | 0.60 |
| **BSREM_400_** | 3.00 | 0.78 | 3.15 | 0.48 | 2.88 | 0.85 | 3.15 | 0.48 | 2.23 | 1.00 | 2.15 | 0.49 | 1.45 | 0.59 | 2.15 | 0.49 |
| **BSREM_500_** | 2.83 | 0.84 | 2.70 | 0.68 | 2.68 | 0.94 | 2.70 | 0.68 | 2.13 | 0.88 | 1.75 | 0.58 | 1.35 | 0.62 | 1.75 | 0.58 |
| **BSREM_600_** | 2.48 | 0.75 | 2.35 | 0.70 | 2.35 | 0.73 | 2.35 | 0.70 | 1.73 | 0.67 | 1.45 | 0.55 | 1.27 | 0.55 | 1.45 | 0.55 |
| **BSREM_700_** | 2.00 | 0.67 | 2.13 | 0.64 | 2.00 | 0.75 | 2.13 | 0.64 | 1.45 | 0.55 | 1.25 | 0.49 | 1.05 | 0.22 | 1.25 | 0.49 |
| **OSEM** | 1.93 | 0.76 | 2.18 | 0.78 | 1.85 | 0.73 | 2.18 | 0.78 | 1.48 | 0.78 | 1.30 | 0.64 | 1.08 | 0.26 | 1.30 | 0.64 |
|  | | | | | | | | | | | | | | | | |
| **Reconstruction** | **Neocortex noise** | | | | **Basal ganglia noise** | | | | **Cerebellum noise** | | | | **Brainstem noise** | | | |
|  | **Reader 1** | | **Reader 2** | | **Reader 1** | | **Reader 2** | | **Reader 1** | | **Reader 2** | | **Reader 1** | | **Reader 2** | |
|  | **Mean** | **SD** | **Mean** | **SD** | **Mean** | **SD** | **Mean** | **SD** | **Mean** | **SD** | **Mean** | **SD** | **Mean** | **SD** | **Mean** | **SD** |
| **BSREM_100_** | 3.90 | 0.49 | 3.90 | 0.49 | 3.70 | 0.60 | 3.90 | 0.49 | 3.60 | 0.77 | 3.90 | 0.49 | 3.73 | 0.75 | 3.90 | 0.49 |
| **BSREM_200_** | 3.18 | 0.78 | 3.40 | 0.84 | 2.98 | 0.73 | 3.40 | 0.84 | 3.00 | 0.71 | 3.40 | 0.84 | 2.95 | 0.84 | 3.40 | 0.84 |
| **BSREM_300_** | 2.20 | 0.82 | 2.75 | 0.95 | 2.00 | 0.81 | 2.75 | 0.95 | 2.05 | 0.93 | 2.75 | 0.95 | 1.88 | 0.96 | 2.75 | 0.95 |
| **BSREM_400_** | 1.50 | 0.67 | 2.00 | 1.08 | 1.38 | 0.62 | 2.00 | 1.08 | 1.48 | 0.84 | 2.00 | 1.08 | 1.40 | 0.74 | 2.00 | 1.08 |
| **BSREM_500_** | 1.13 | 0.46 | 1.40 | 0.81 | 1.10 | 0.37 | 1.40 | 0.81 | 1.15 | 0.42 | 1.40 | 0.81 | 1.15 | 0.42 | 1.40 | 0.81 |
| **BSREM_600_** | 1.05 | 0.22 | 1.03 | 0.15 | 1.13 | 0.51 | 1.03 | 0.15 | 1.02 | 0.15 | 1.03 | 0.15 | 1.05 | 0.22 | 1.03 | 0.15 |
| **BSREM_700_** | 1.00 | 0.00 | 1.03 | 0.15 | 1.00 | 0.00 | 1.03 | 0.15 | 1.02 | 0.15 | 1.03 | 0.15 | 1.00 | 0.00 | 1.03 | 0.15 |
| **OSEM** | 1.15 | 0.53 | 1.23 | 0.73 | 1.18 | 0.59 | 1.23 | 0.73 | 1.05 | 0.22 | 1.23 | 0.73 | 1.00 | 0.00 | 1.23 | 0.73 |
| ***Note****: BSREM = block sequential regularized maximization. GIq =* general image quality. *OSEM = ordered subset expectation maximization* | | | | | | | | | | | | | | | | |

**Table S3**. Quadratic weighted Cohen’s kappa (k) statistic of the agreement between reader 1 and reader 2.

| **Reconstruction** | **General image quality** | | **Noise score** | | | **Lesion detectability** | | |
| --- | --- | --- | --- | --- | --- | --- | --- | --- |
|  | **Quadratic weighted k C.I.)** | **Proportions of Agreement observed** | **Quadratic weighted k (C.I.)** | **Proportions of Agreement observed** | | **Quadratic weighted k (C.I.)** | | **Proportions of Agreement observed** |
| **BSREM_100_** | 0.055 (0.00-0.12) | 25% | 0.790 (0.54-1.00) | 95% | | 0.513 (0.29-0.73) | | 40% |
| **BSREM_200_** | 0.268 (0.09-0.44) | 65% | 0.560 (0.20-0.92) | 52% | | 0.708 (0.68-0.73) | | 47% |
| **BSREM_300_** | 0.047 (0.00-0.33) | 45% | 0.230 (0.01-0.28) | 17% | | 0.661 (0.45-0.75) | | 55% |
| **BSREM_400_** | < 0.000 | 45% | 0.356 (0.08-0.62) | 52% | | 0.750 (0.66-0.83) | | 57% |
| **BSREM_500_** | < 0.000 | 35% | 0.152 (0.00-0.38) | 80% | | 0.701 (0.53-0.80) | | 57% |
| **BSREM_600_** | < 0.000 | 37% | 0.655 (0.13-1.00) | 97% | | 0.730 (0.55-0.82) | | 62% |
| **BSREM_700_** | < 0.000 | 42% | 1.000 (1.00-1.00) | 100% | | 0.730 (0.55-0.82) | | 62% |
| **OSEM** | 0.306 (0.12-0.48) | 52% | 0.802 (0.67-0.93) | 90% | | 0.689 (0.61-0.76) | | 50% |
|  | | | | | | | | |
| **Reconstruction** | **Hypermetabolic metastases** | | **Hypometabolic metastases** | | | **Edema** | | |
|  | **Quadratic weighted k (C.I.)** | **Proportions of Agreement observed** | **Quadratic weighted k (C.I.)** | **Proportions of Agreement observed** | | **Quadratic weighted k (C.I.)** | | **Proportions of Agreement observed** |
| **BSREM_100_** | 0.518 (0.31-0.71) | 75% | 1.000 (1.00-1.00) | 100% | | 0.514 (0.31-0.71) | | 75% |
| **BSREM_200_** | 0.561 (0.36-0.75) | 77% | 1.000 (1.00-1.00) | 100% | | 0.514 (0.31-0.71) | | 75% |
| **BSREM_300_** | 0.561 (0.36-0.75) | 77% | 1.000 (1.00-1.00) | 100% | | 0.514 (0.31-0.71) | | 75% |
| **BSREM_400_** | 0.608 (0.41-0.80) | 80% | 1.000 (1.00-1.00) | 100% | | 0.644 (0.45-0.83) | | 82% |
| **BSREM_500_** | 0.500 (0.23-0.76) | 75% | 1.000 (1.00-1.00) | 100% | | 0.560 (0.33-0.78) | | 80% |
| **BSREM_600_** | 0.506 (0.29-0.71) | 75% | 0.920 (0.80-1.00) | 97% | | 0.670 (0.46-0.87) | | 85% |
| **BSREM_700_** | 0.550 (0.34-0.75) | 77% | 0.750 (0.54-0.96) | 92% | | 0.832 (0.65-1.00) | | 92% |
| **OSEM** | 0.600 (0.39-0.80) | 80% | 1.000 (1.00-1.00) | 100% | | 0.720 (0.53-0.90) | | 87% |
|  | | | | | | | | |
| **Reconstruction** | | **Mass effect** | | | **Metastases blurring** | | | |
|  |  | **Quadratic weighted k (C.I.)** | **Proportions of Agreement observed** | | **Quadratic weighted k (C.I.)** | | **Proportions of Agreement observed** | |
| **BSREM_100_** | | 0.343 (0.07-0.61) | 77% | | 0.908 (0.76-1.00) | | 97% | |
| **BSREM_200_** | | 0.343 (0.07-0.61) | 77% | | 0.754 (0.53-0.97) | | 92% | |
| **BSREM_300_** | | 0.343 (0.07-0.61) | 77% | | 0.688 (0.45-0.92) | | 90% | |
| **BSREM_400_** | | 0.343 (0.07-0.61) | 77% | | 0.688 (0.45-0.92) | | 90% | |
| **BSREM_500_** | | 0.268 (0.00-0.56) | 77% | | 0.844 (0.67-1.00) | | 95% | |
| **BSREM_600_** | | 0.384 (0.10-0.66) | 80% | | 0.778 (0.58-0.97) | | 92% | |
| **BSREM_700_** | | 0.431 (0.14-0.71) | 82% | | 0.692 (0.46-0.91) | | 90% | |
| **OSEM** | | 0.431 (0.14-0.71) | 82% | | 0.655 (0.39-0.91) | | 91% | |
| ***Note****: BSREM = block sequential regularized maximization. GIq =* general image quality. *OSEM = ordered subset expectation maximization* | | | | | | | | |

**Table S4.** Mean differences of PET parameters (brain metastases SUVmax, background SUVmean and TBR) using different reconstruction algorithms are displayed in the upper right half of the table. The lower left half of the table shows *p values* of multiple comparisons of different reconstructions.

﻿

| **Reconstruction** | **Difference of brain metastases SUVmax*** | | | | | | | |
| --- | --- | --- | --- | --- | --- | --- | --- | --- |
|  | **BSREM_100_** | **BSREM_200_** | **BSREM_300_** | **BSREM_400_** | **BSREM_500_** | **BSREM_600_** | **BSREM_700_** | **OSEM** |
| **BSREM_100_** | - | 1.936 | 2.892 | 3.522 | 3.974 | 4.316 | 4.586 | 4.739 |
| **BSREM_200_** | *1.000* | - | 0.956 | 1.586 | 2.038 | 2.381 | 2.651 | 2.804 |
| **BSREM_300_** | *0.339* | *1.000* | - | 0.630 | 1.083 | 1.425 | 1.695 | 1.848 |
| **BSREM_400_** | *0.066* | *1.000* | *1.000* | - | 0.453 | 0.795 | 1.065 | 1.218 |
| **BSREM_500_** | *0.017* | *1.000* | *1.000* | *1.000* | - | 0.342 | 0.612 | 0.765 |
| **BSREM_600_** | *0.006* | *1.000* | *1.000* | *1.000* | *1.000* | - | 0.270 | 0.423 |
| **BSREM_700_** | *0.002* | *0.599* | *1.000* | *1.000* | *1.000* | *1.000* | - | 0.153 |
| **OSEM** | *0.002* | *0.420* | *1.000* | *1.000* | *1.000* | *1.000* | *1.000* | - |
|  | | | | | | | | |
| **Reconstruction** | **Difference of background SUVmean (neocortex)*** | | | | | | | |
|  | **BSREM_100_** | **BSREM_200_** | **BSREM_300_** | **BSREM_400_** | **BSREM_500_** | **BSREM_600_** | **BSREM_700_** | **OSEM** |
| **BSREM_100_** | - | 0.041 | 0.041 | 0.041 | -0.043 | -0.116 | -0.083 | 0.0005 |
| **BSREM_200_** | *1.000* | - | -0.0008 | -0.0005 | -0.084 | -0.158 | -0.124 | -0.041 |
| **BSREM_300_** | *1.000* | *1.000* | - | 0.0002 | -0.084 | -0.157 | -0.124 | -0.040 |
| **BSREM_400_** | *1.000* | *1.000* | *1.000* | - | -0.084 | -0.157 | -0.124 | -0.040 |
| **BSREM_500_** | *1.000* | *1.000* | *1.000* | *1.000* | - | -0.073 | -0.040 | 0.043 |
| **BSREM_600_** | *1.000* | *1.000* | *1.000* | *1.000* | *1.000* | - | 0.033 | 0.116 |
| **BSREM_700_** | *1.000* | *1.000* | *1.000* | *1.000* | *1.000* | *1.000* | - | 0.083 |
| **OSEM** | *1.000* | *1.000* | *1.000* | *1.000* | *1.000* | *1.000* | *1.000* | - |
|  | | | | | | | | |
| **Reconstruction** | **Difference of TBR*** | | | | | | | |
|  | **BSREM_100_** | **BSREM_200_** | **BSREM_300_** | **BSREM_400_** | **BSREM_500_** | **BSREM_600_** | **BSREM_700_** | **OSEM** |
| **BSREM_100_** | - | 0.341 | 0.515 | 0.628 | 0.738 | 0.811 | 0.883 | 0.847 |
| **BSREM_200_** | *1.000* | - | 0.175 | 0.287 | 0.397 | 0.471 | 0.499 | 0.507 |
| **BSREM_300_** | *0.114* | *1.000* | - | 0.113 | 0.223 | 0.296 | 0.324 | 0.332 |
| **BSREM_400_** | *0.014* | *1.000* | *1.000* | - | 0.110 | 0.184 | 0.212 | 0.220 |
| **BSREM_500_** | *0.001* | *0.737* | *1.000* | *1.000* | - | 0.073 | 0.101 | 0.109 |
| **BSREM_600_** | *0.0001* | *0.241* | *1.000* | *1.000* | *1.000* | - | 0.028 | 0.036 |
| **BSREM_700_** | *0.0001* | *0.152* | *1.000* | *1.000* | *1.000* | *1.000* | - | 0.008 |
| **OSEM** | *0.0001* | *0.132* | *1.000* | *1.000* | *1.000* | *1.000* | *1.000* | - |
| ********Calculated as (SUVmax in each dataset – SUVmax reference) x 100 / (SUVmax reference), where dataset reconstructions are listed in the row and reference reconstruction are listed in the column.*  ***Statistically significant based on Bonferroni adjustment to multiple comparison.*  ***Note****: BSREM = block sequential regularized maximization, OSEM = ordered subset expectation maximization, TBR = target-to-background ratio.* | | | | | | | | |

**Table S5**. Differences in brain metastases’ SUVmax (expressed as *p* values) between different reconstructions, stratified by BMI, MBq/kg, glucose, brain metastasis size.

| **Metastases’ SUVmax** | **BSREM100** | **BSREM200** | **BSREM300** | **BSREM400** | **BSREM500** | **BSREM600** | **BSREM700** | **OSEM** |
| --- | --- | --- | --- | --- | --- | --- | --- | --- |
|  | ***p value**** | ***p value**** | ***p value**** | ***p value**** | ***p value**** | ***p value**** | ***p value**** | ***p value**** |
| **BMI >25** | 0.185 | 0.143 | 0.108 | 0.086 | 0.069 | 0.076 | 0.063 | **0.049** |
| **MBq per kg <2** | 0.749 | 0.855 | 0.738 | 0.648 | 0.563 | 0.523 | 0.484 | 0.584 |
| **Glucose >5.5 mmol/l** | 0.234 | 0.183 | 0.157 | 0.149 | 0.161 | 0.183 | 0.202 | 0.095 |
| **Lesion >1.5 cm**** | 0.876 | 0.950 | 0.901 | 0.827 | 0.901 | 0.975 | 0.950 | 0.851 |
| **Note**: BMI = body mass index  **p-value* was calculated with Mann Whitney U-test  **lesion size was obtained on MRI, measured by the longest in-plane diameter of the lesion. | | | | | | | | |

﻿

**SUPPLEMENTAL MATERIAL - FIGURES AND FIGURES LEGENDS**

**Figure S1**. Flow chart of patient inclusion / exclusion.


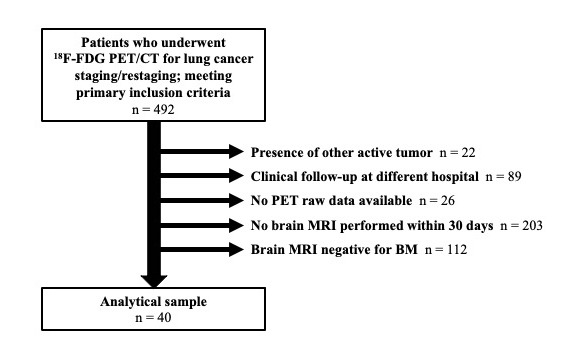


**Figure S2.** Exemplary case of a 47-year-old patient with a staging ^18^F-FDG PET/CT performed for NSCLC. The MR images performed 4 days after PET/CT show several brain metastases, in particular one partially solid contrast-enhancing and partially necrotic metastasis in the left cerebellum (white arrows). In PET/CT images, this lesion corresponds to a strongly photopenic area (black arrows), easily detectable in all reconstructions, regardless of blurring.

*
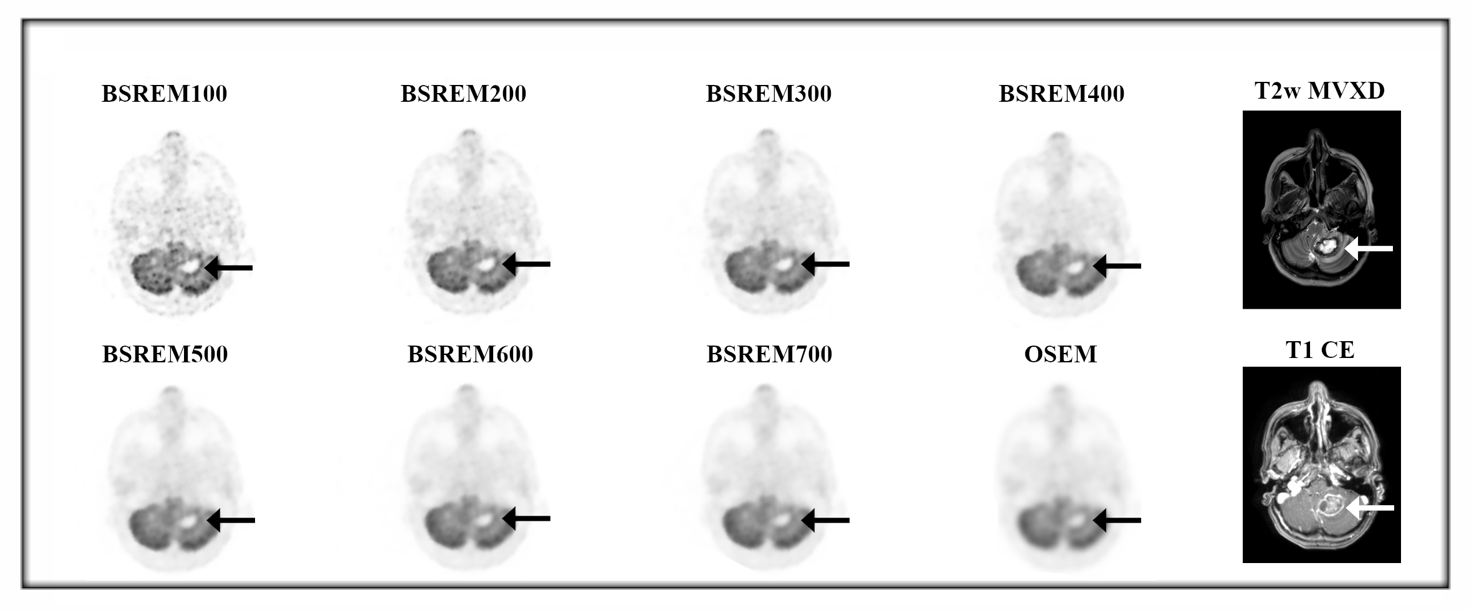
*

**Figure S3.** Box plot of PET parameters measured by reader 2, using cloned VOIs for all reconstructions: brain metastases SUVmax (A), target-to-background ratio (TBR) (B), contrast recovery (CR) ratio comparing each reconstruction with OSEM as reference (C) and CR ratio comparing each reconstruction with BSREM_400_ as reference (D). The boxes indicate mean and standard deviation over subjects, and the whiskers cover the range from minimum to maximum, asterisks and dots denote outliers.


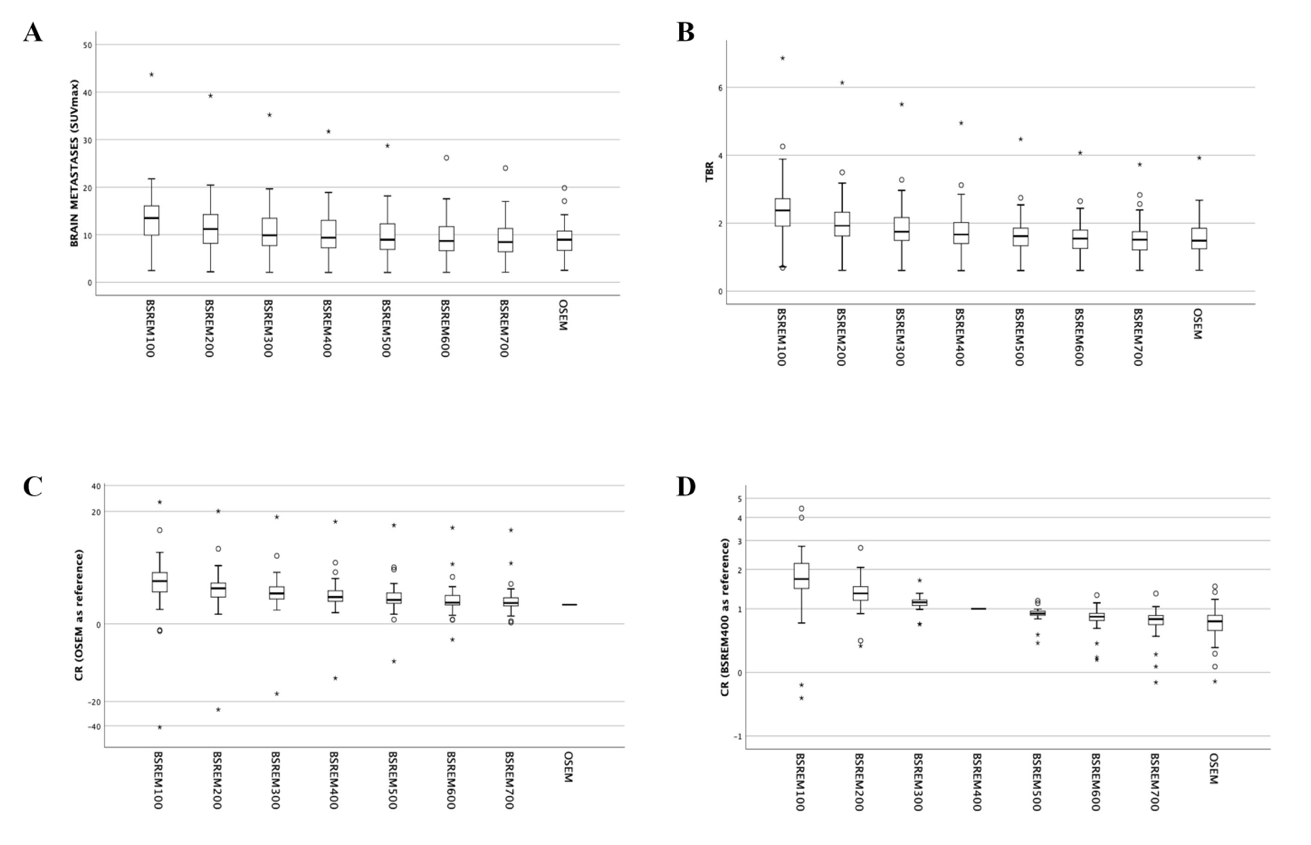


**Figure S4.** Exemplary case of a 70-year-old patient with a staging ^18^F-FDG PET/CT. The PET/CT scan shows two brain metastases (black arrows): one in the left paracentral lobe (1) and one in the left temporal lobe (2), respectively. Lesions are also seen on the T1-weighted turbo field echo (TFE) contrast-enhanced (ce) MR images (white arrows on both coronal (a) and axial (b) images). Both brain metastases are characterized by high FDG-avidity, rendering them well appreciated in all PET reconstructions. The SUVmax of both lesions decreased considerably from BSREM_100_ to BSREM_700_ (inversely to the increase of the β-value) and OSEM. However, despite significantly higher SUVmax, BSREM_100_ was not characterized by better lesion detection, especially for the one in the left paracentral lobe (1), due to high noise impeding tumor detectability. On the other hand, BSREM with higher β-value (as in this case BSREM_700_) and OSEM reconstructions are characterized by more blurring, leading to a loss of accuracy in detectability of small and less FDG-avid lesion, being more evident for the central-necrotic metastasis in the left temporal lobe (2).


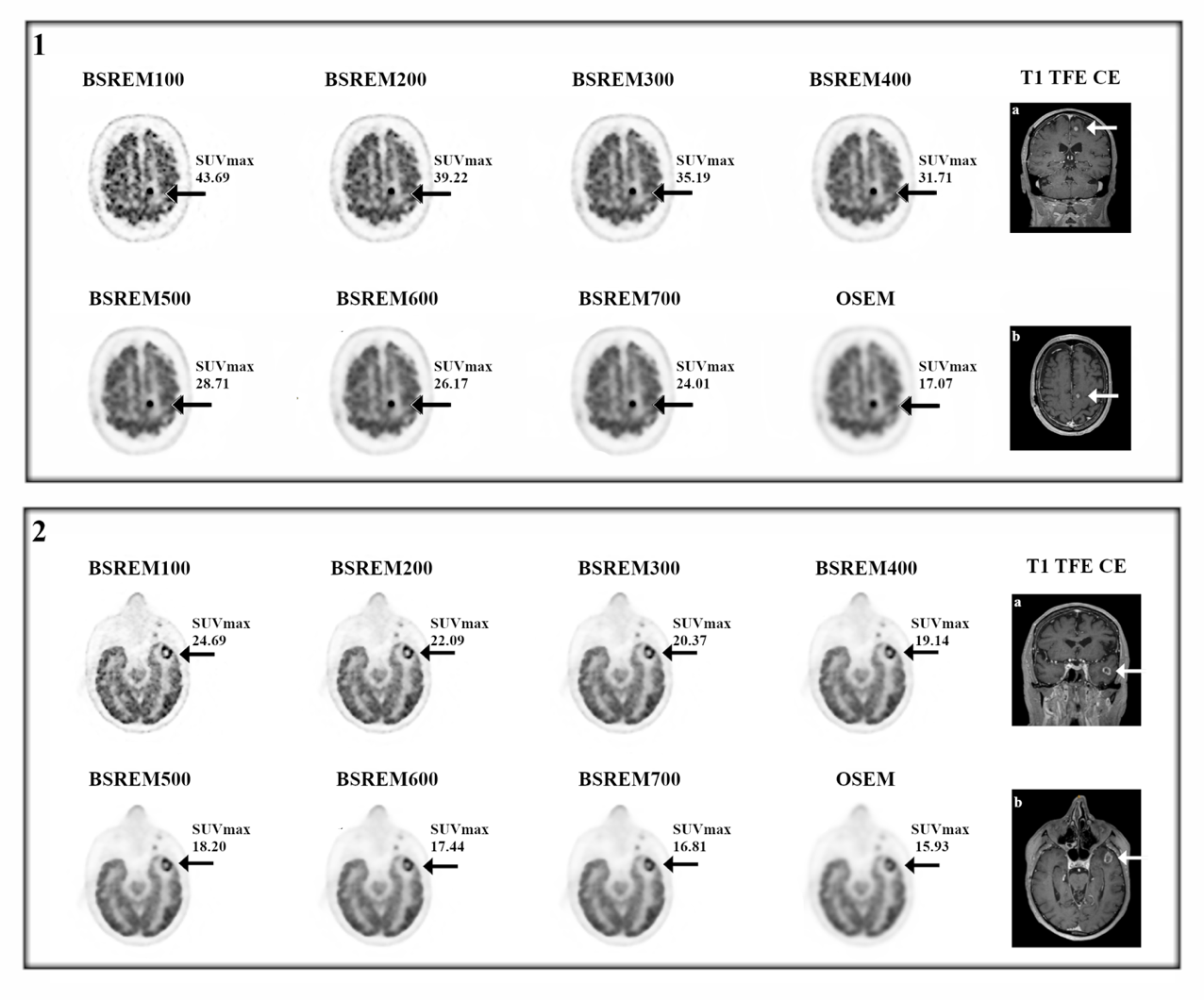

Supplement: Supplementary file 1 — Supplementary file1 (DOCX 889 KB) [file 10278_2021_570_MOESM1_ESM.docx]
