# Supplementary figures and images for: BSREM for Brain Metastasis Detection with 18F-FDG-PET/CT in Lung Cancer Patients
Source: J Digit Imaging. 2022 Feb 25;35(3):581–93. doi: 10.1007/s10278-021-00570-y (PMC9156589; doi:10.1007/s10278-021-00570-y)

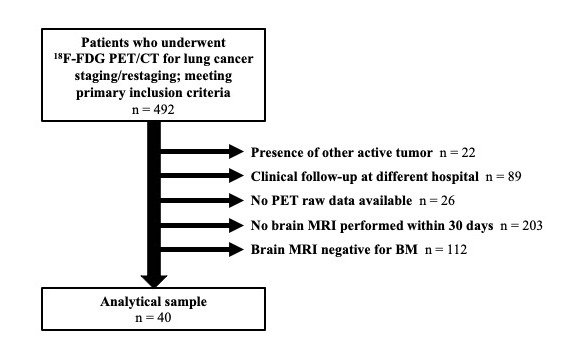

Supplement: Supplementary file 2 — Supplementary file2 (JPG 37 KB) [file 10278_2021_570_MOESM2_ESM.jpg]

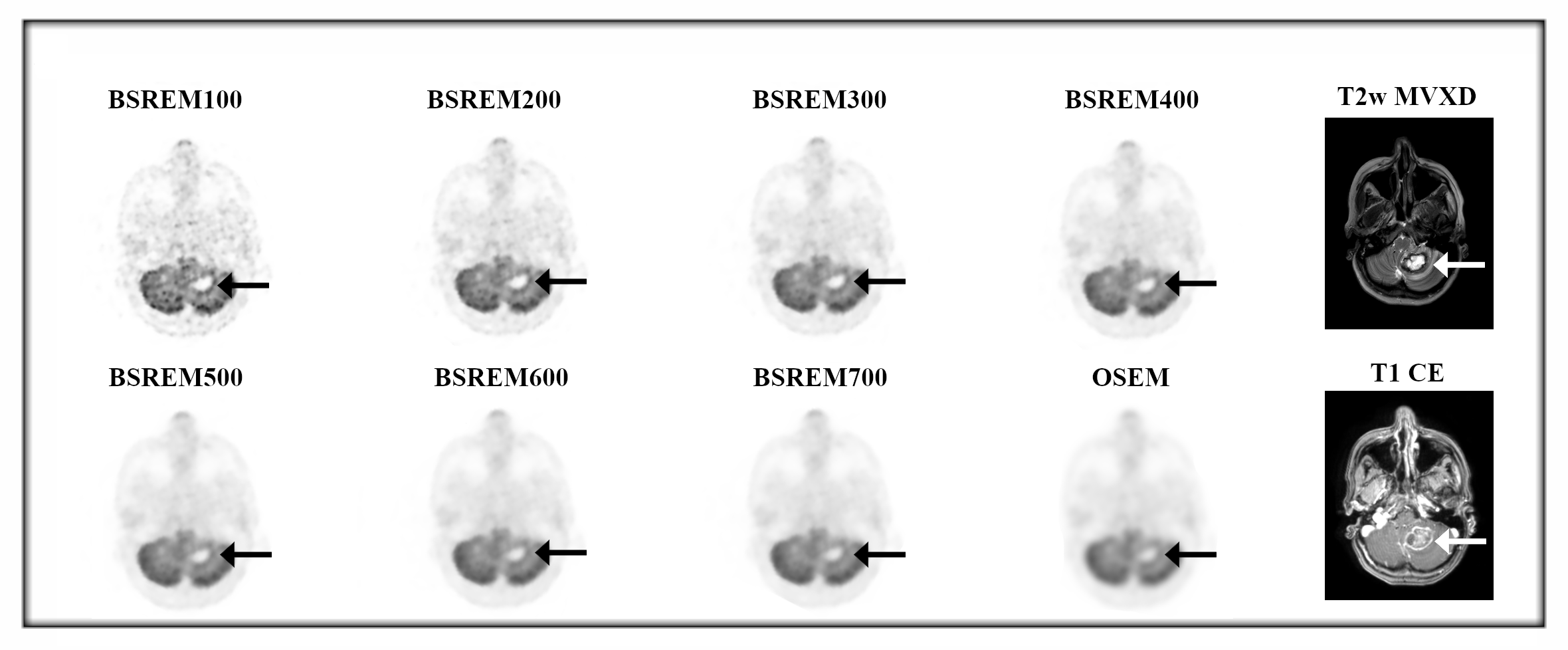

Supplement: Supplementary file 3 — Supplementary file3 (PNG 367 KB) [file 10278_2021_570_MOESM3_ESM.png]

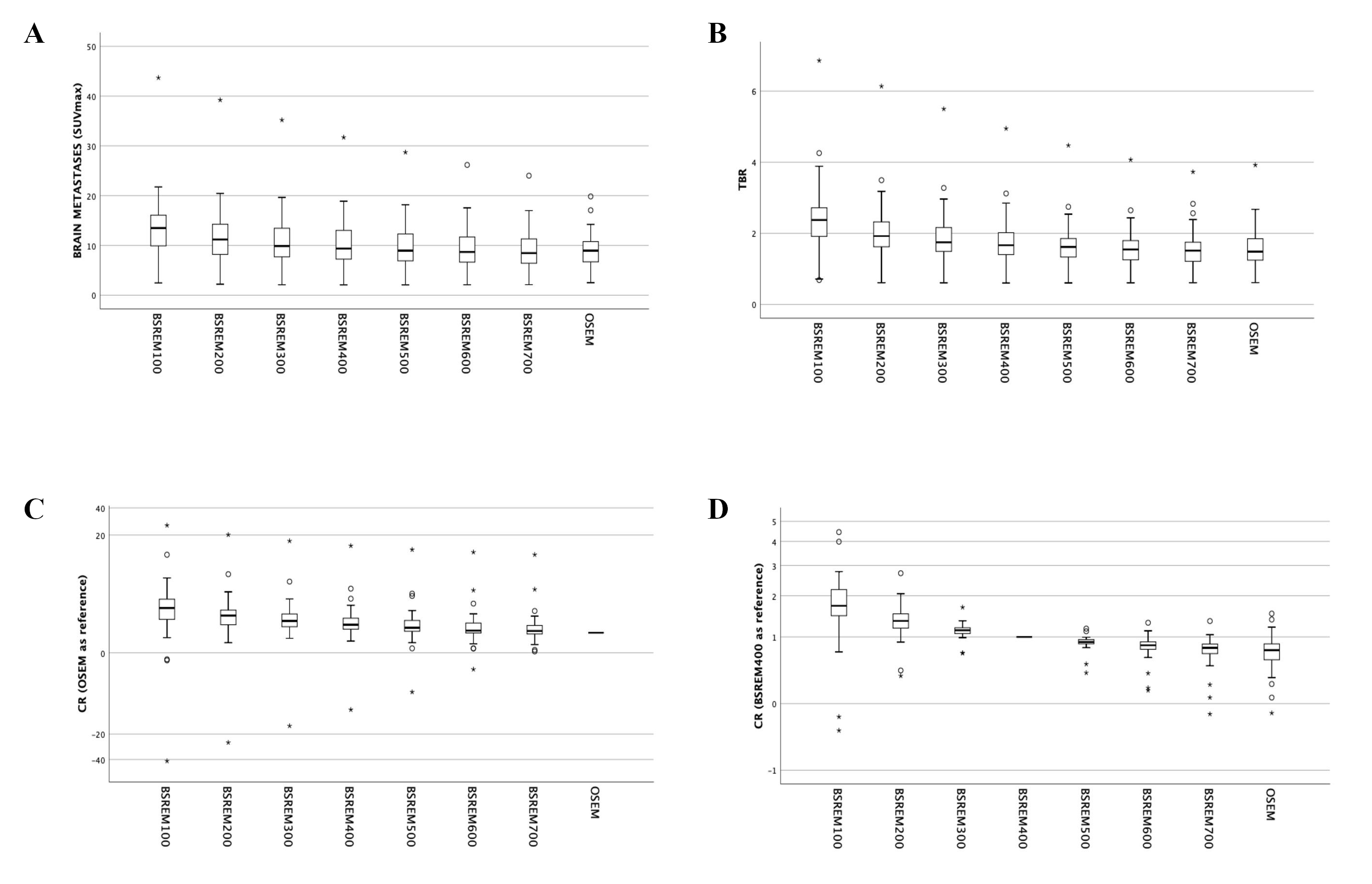

Supplement: Supplementary file 4 — Supplementary file4 (PNG 491 KB) [file 10278_2021_570_MOESM4_ESM.png]

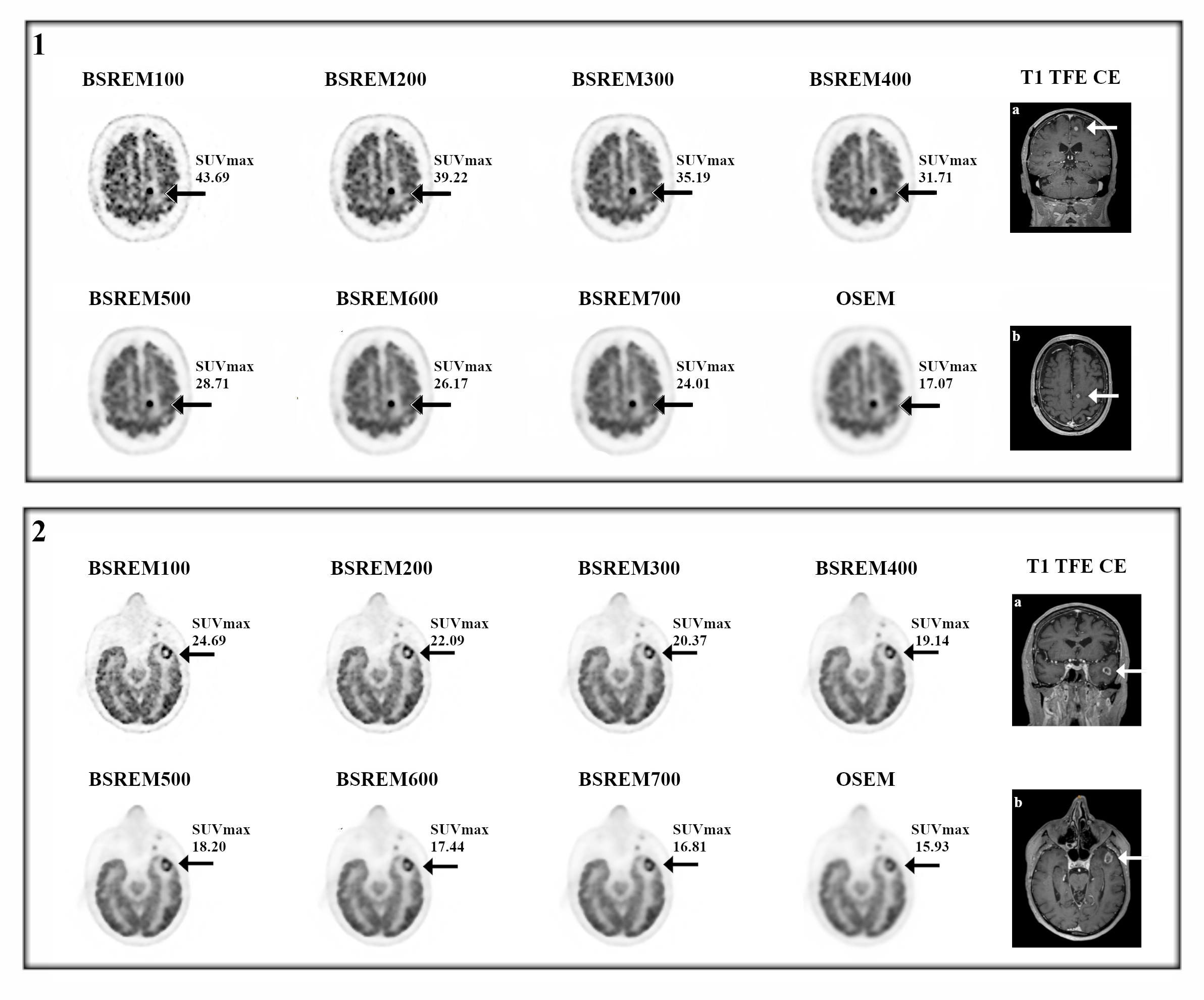

Supplement: Supplementary file 5 — Supplementary file5 (PNG 819 KB) [file 10278_2021_570_MOESM5_ESM.png]
